# Painful sex (dyspareunia) in women: prevalence and associated factors in a British population probability survey

**DOI:** 10.1111/1471-0528.14518

**Published:** 2017-01-25

**Authors:** KR Mitchell, R Geary, CA Graham, J Datta, K Wellings, P Sonnenberg, N Field, D Nunns, J Bancroft, KG Jones, AM Johnson, CH Mercer

**Affiliations:** ^1^ Centre for Sexual and Reproductive Health Research Department of Social and Environmental Health Research London School of Hygiene and Tropical Medicine London UK; ^2^ MRC/CSO Social and Public Health Sciences Unit University of Glasgow Glasgow UK; ^3^ Centre for Sexual Health and HIV Research Research Department of Infection & Population Health University College London London UK; ^4^ Centre for Sexual Health Research Department of Psychology University of Southampton Southampton UK; ^5^ Department of Gynaecology Nottingham University Hospitals NHS Trust Nottingham UK; ^6^ Department of Psychiatry University of Oxford Oxford UK

**Keywords:** Britain, co‐morbidity, dyspareunia, general population survey, prevalence, sexual dysfunction, sexual function problems, sexual relationship, UK

## Abstract

**Objective:**

To estimate the prevalence of painful sex among women in Britain, and to explore associated sexual, relationship and health factors that should be considered in assessment.

**Design:**

Multi‐stage, clustered and stratified population probability sample survey, using computer‐assisted self‐interview. Sample frame was the British Postcode Address File.

**Setting:**

Participants interviewed at home between 2010 and 2012.

**Sample:**

A total of 15 162 adults aged 16–74 years (8869 women). Data reported from 6669 sexually active women.

**Methods:**

Age‐adjusted logistic regressions to examine associations between painful sex and indicators of sexual, relational, mental and physical health.

**Main outcome measure:**

Physical pain as a result of sex for ≥3 months in the past year, plus measures of symptom severity.

**Results:**

Painful sex was reported by 7.5% (95% CI 6.7–8.3) of sexually active women, of whom one‐quarter experienced symptoms very often or always, for ≥6 months, and causing distress. Reporting painful sex was strongly associated with other sexual function problems, notably vaginal dryness (age adjusted odds ratio 7.9; 6.17–10.12), anxiety about sex (6.34; 4.76–8.46) and lacking enjoyment in sex (6.12; 4.81–7.79). It was associated with sexual relationship factors [such as not sharing same level of interest in sex (2.56; 1.97–3.33)], as well as with adverse experiences such as non‐volitional sex (2.17; 1.68–2.80). Associations were also found with measures of psychological and physical health, including depressive symptoms (1.68; 1.28–2.21).

**Conclusion:**

Painful sex is reported by a sizeable minority of women in Britain. Health professionals should be supported to undertake holistic assessment and treatment which takes account of the sexual, relationship and health context of symptoms.

**Tweetable abstract:**

Painful sex—reported by 7.5% of women in Britain—is linked to poorer sexual, physical, relational and mental health.

## Tweetable abstract

Painful sex–reported by 7.5% of women in Britain–is linked to poorer sexual, physical, relational and mental health.

This article includes Author Insights, a video abstract available at https://vimeo.com/rcog/authorinsights14518.

## Graphical abstract

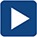

## Introduction

Painful sex (dyspareunia) is a common but neglected female health problem.^1^ The population prevalence is estimated to vary from 3 to 18% globally,^2^ and lifetime estimates range from 10 to 28%.^3^ Wide ranges reflect significant heterogeneity in methodologies of prevalence studies.^4^ The underlying conditions are often difficult to diagnose and treat,^2^, ^5^, ^6^ and the aetiological factors are complex, and poorly understood. Partly because of this, sexual pain disorders are often overlooked or badly managed, significantly exacerbating patient distress.^7^ There are many potential causes, and efforts to understand and characterise the problem are hampered by classificatory challenges^1^, ^8^ and scant empirical evidence.^9^ Subtyping into deep (pain felt within pelvis) and superficial (pain felt in vulval area) is common,^9^ but may undermine attempts to treat the experience of pain holistically. In the most recent version of the Diagnostic and Statistical Manual of Mental Disorders^10^ dyspareunia and vaginismus (involuntary spasm of musculature of the vagina) were deleted, and a new diagnosis of Genito‐pelvic pain/penetration disorder was introduced.

Dyspareunia is a common and troubling complaint of women with vulvodynia, and in particular provoked vestibulodynia. Painful sex can also result from a range of conditions causing genital pain, including vulval skin conditions (e.g. lichen sclerosus), vulvovaginal and urinary tract infections, sexually transmitted infections (STIs) and endometriosis.^11^ Dyspareunia is often co‐morbid with sexual difficulties such as lack of desire and arousal,^12^ and strain within the sexual relationship.^13^ Psychosocial correlates include negative body image, catastrophising, hypervigilance to pain, depression and anxiety, and low self‐esteem.^12^, ^14^, ^15^, ^16^ Although these psychosocial variables may be both aetiological and sequelae, the cross‐sectional nature of most studies limits understanding of causal direction.^8^

To date, most research on painful sex has been based on small clinical samples.^5^, ^12^ These studies exclude women who have not sought help^3^ and are therefore not representative of the general population. Community surveys are limited,^5^, ^17^ as are studies that investigate medical and psychosocial factors simultaneously. Specific subgroups, such as adolescent women, have also received little focused research attention.^18^ The objective of this study is to estimate the population prevalence of painful sex in women and to address the gap in data describing the health, relationship and sexual context in which painful sex is experienced. To do this we use data from the third National Survey of Sexual Attitudes and Lifestyles; a large probability survey undertaken in Britain.

## Methods

### Participants and procedure

We present data from women aged 16–74 years who participated in Natsal‐3, a multi‐stage, clustered, stratified probability sample survey of 15 162 adults (8869 women) in Britain, interviewed between September 2010 and August 2012. This paper is focused on sexually active women, defined as those reporting vaginal, oral or anal sex with one or more partners of either gender in the past year. We also report briefly on sexually inactive women, defined as having sexual experience, but no sex in the past year (small numbers precluded detailed analysis of this group).

Participants were interviewed at home using a combination of computer‐assisted face‐to‐face and self‐interview for the more sensitive questions. The survey instrument underwent thorough cognitive testing and piloting.^19^ After weighting to adjust for unequal probabilities of selection, the Natsal‐3 sample was broadly representative of the British population as described by 2011 Census figures.^20^

The estimated response rate for Natsal‐3 was 57.7%, and the co‐operation rate was 65.8% (of all eligible addresses contacted). Details of the survey methodology are published elsewhere.^20^, ^21^ Natsal‐3 was approved by the NRES Committee South Central—Oxford A (Ref: 10/H0604/27). Participants provided oral informed consent for interviews.

### Measures

Women who were sexually active in the last year were asked whether they had experienced any of a list of eight difficulties with their sex life lasting ≥3 months in the past year, including ‘felt physical pain, as a result of sex’ (painful sex). (The eight difficulties were: lacked interest in sex, lacked enjoyment in sex, felt anxious during sex, felt physical pain as a result of sex, felt no excitement or arousal during sex, did not reach a climax or took a long time to reach a climax despite feeling excited or aroused, reached climax more quickly than you would like, and had an uncomfortably dry vagina.) Women reporting painful sex were asked how long they had experienced this, how often symptoms occurred, and how they felt about it. ‘Morbid pain’ was defined as symptoms experienced for ≥6 months, symptoms occurring very often or always, and participant fairly or very distressed about the difficulty (morbid painful sex). Women who had ever had a sexual experience, but were not sexually active in the last year were asked if they had avoided sex because of sexual difficulties (either their own or those of their partner). Those who answered yes were asked to indicate the reasons, with options including ‘felt physical pain, or feared feeling physical pain, as a result of sex’.

Among sexually active women, we tested for associations between reporting painful sex and a range of explanatory variables, selected to reflect the health, relationship and sexual context of women's lives and to identify factors potentially relevant to clinical assessment and management.

### Statistical analysis

All analyses were carried out using the complex survey functions of STATA (version 14; StataCorp LP, College Station, TX, USA) to account for the weighting, clustering and stratification of the data. We present descriptive statistics for reporting of painful sex and morbid painful sex. We used age‐adjusted logistic regressions to examine the associations between reporting physical pain during sex and other sexual function problems, demographic and health factors, sexual behaviour, sexual relationship and attitudes to sex. We performed a sensitivity analysis to check whether the observed associations differed according to age (16–44 years age group versus 45–74 years). Few differences were observed, so we did not present the results stratified by age.

## Results

### Prevalence of painful sex and morbid painful sex

Table 1 shows the prevalence of reporting painful sex and morbid painful sex by age group. Among sexually active women aged 16–74 years, 7.5% (95% CI 6.7–8.3) reported painful sex; 4.6% (4.1–5.3) reported symptoms lasting ≥6 months, of whom around half were distressed about their symptoms. <2% (1.9%; 1.5–2.3) of all sexually active women reported morbid painful sex (i.e. met all three morbidity criteria). The proportion reporting painful sex was highest in the youngest women (16–24 years) and those aged 55–64 years, whereas morbid pain was highest (3.9%) in this latter age group.

**Table 1:** Prevalence of reporting painful sex and morbid painful sex by age group among sexually active women aged 16–74 years

		Pain during sex for 3 months or more, past year		Pain during sex for 6 months or more, past year		Pain during sex for 6 months or more and distress about this, past year		Morbid pain: pain during sex for 6 months or more, distress about this, and symptoms frequent^a^	
	Denominators Unw., wtd^b^	%	95% CI	%	95% CI	%	95% CI	%	95% CI
**All women**	6669, 5755	7.5	(6.7–8.3)	4.6	(4.1–5.3)	2.5	(2.1–3.0)	1.9	(1.5–2.3)
**Age group**									
16–24	1662, 923	9.5	(8.1–11.2)	3.8	(3.0–5.0)	2.1	(1.5–3.0)	1.7	(1.1–2.5)
25–34	2236, 1246	8.0	(6.7–9.4)	4.6	(3.6–5.7)	2.7	(2.0–3.6)	2.0	(1.4–2.8)
35–44	1050, 1290	5.2	(3.9–7.0)	3.4	(2.4–4.9)	2.2	(1.4–3.4)	1.5	(0.9–2.5)
45–54	871, 1186	6.4	(4.9–8.5)	3.9	(2.7–5.6)	2.1	(1.3–3.4)	1.2	(0.6–2.1)
55–64	569, 755	10.4	(7.9–13.4)	8.8	(6.6–11.7)	4.3	(2.7–6.8)	3.9	(2.4–6.2)
65–74	281, 355	5.3	(3.2–8.8)	5.0	(2.9–8.3)	1.8	(0.7–4.2)	1.1	(0.4–3.1)

a Specific criteria: symptoms experienced for at least 6 months, participant fairly or very distressed about the difficulty and symptoms occurring very often or always.

b Unw., unweighted; wtd, weighted.

Among women aged 16–74 years, 1505 were not sexually active in the last year and answered the item on avoidance of sex because of sexual difficulties. Of these, 211 (17.23%) reported avoiding sex, with 35 reporting pain, or fear of feeling pain, as a reason for avoidance (2.49% of all sexually inactive women). [Correction added on 7 April 2017, after first online publication: The number of non‐sexually active participants has been changed from 1708 to 1505, and therefore the percentage calculations have been corrected in the preceeding sentence.]

### Association of painful sex with sexual function problems and sexual satisfaction

Many women reporting painful sex also reported another sexual function problem (Table 2): 62.0% reported lack of interest in sex (compared with 31.9% of women with no pain), 45.2% reported vaginal dryness (compared with 10.4%), 40.2% reported difficulty reaching climax (compared with 14.4%), and 40.1% reported lacking enjoyment (compared with 9.9%). Painful sex was strongly associated with all the sexual function problems we measured, and in particular, with vaginal dryness [age adjusted odds ratio (aOR) 7.9; 95% CI 6.17–10.12], feeling anxious during sex (aOR 6.34; 95% CI 4.76–8.46) and lacking enjoyment in sex (aOR 6.12; 95% CI 4.81–7.79).

**Table 2:** Association of painful sex (lasting ≥3 months in the last year) with sexual enjoyment, sexual function and satisfaction, among sexually active women

Denominators (unweighted, weighted)	Women NOT reporting painful sex^a^		Women reporting painful sex		Age‐adjusted logistic regression		
	6163, 5327		506, 429				
	%	95% CI	%	95% CI	aOR	95% CI	*P*‐value
Lacked interest in having sex	31.90	(30.5–33.3)	62.00	(56.8–66.9)	3.55	(2.83–4.46)	<0.0001
Lacked enjoyment in sex	9.90	(9.0–10.8)	40.10	(34.9–45.6)	6.12	(4.81–7.79)	<0.0001
No excitement/arousal during sex	6.90	(6.2–7.6)	24.30	(19.9–29.5)	4.35	(3.29–5.74)	<0.0001
Felt anxious during sex	4.00	(3.5–4.6)	20.70	(16.9–25.2)	6.34	(4.76–8.46)	<0.0001
Uncomfortably dry vagina	10.40	(9.4–11.4)	45.20	(40.0–50.4)	7.90	(6.17–10.12)	<0.0001
Difficultly in reaching climax	14.40	(13.5–15.4)	40.20	(35.2–45.5)	4.00	(3.19–5.02)	<0.0001
Dissatisfied with sex life	10.10	(9.3–11.1)	30.90	(26.1–36.2)	4.00	(3.09–5.16)	<0.0001
Distressed or worried about sex life	9.20	(8.4–10.1)	28.90	(24.3–34.1)	4.02	(3.11–5.21)	<0.0001
Avoided sex because of own or partner's sexual difficulties	10.70	(9.8–11.7)	44.90	(39.6–50.4)	7.22	(5.66–9.20)	<0.0001

a Painful sex lasting ≥3 months in last year (no morbidity criteria applied).

Just under a third of women reporting painful sex said they were dissatisfied with their sex life (30.9%; compared with 10.1% of women not reporting painful sex; aOR 4.00; 95% CI 3.09–5.16) and 28.9% said they were distressed or worried about their sex life (compared with 9.2% of women not reporting painful sex; aOR 4.02; 95% CI 3.11–5.21). Women reporting painful sex were much more likely to say that they had avoided sex in the past year because of sexual difficulties (own or those of partner) (44.9 versus 10.7% of those not reporting pain; aOR 7.22; 95% CI 5.66–9.20).

### Association of painful sex with socio‐demographic, general health, and sexual lifestyles and attitudes

Table 3 shows associations between reporting painful sex (lasting ≥3 months) and selected socio‐demographic and health factors. After adjusting for age, reporting painful sex was associated with higher educational attainment (aOR for further academic qualifications versus none 2.10; 1.39–3.16), and with being retired (aOR for retired versus full‐time employed 1.79; 1.10–2.90).

**Table 3:** Socio‐demographic and health factors associated with reporting painful sex lasting ≥3 months in the last year, among sexually active women aged 16–74 years

	Denominators Unw, wtd^a^	Pain during sex for 3 months or more, past year				
		%	95% CI	aOR	95% CI	*P*‐value
**All women**	6669, 5755	7.50	(6.7–8.3)			
***Socio–demographic factors***						
**Quintile of Index of Multiple Deprivation** b						
[least deprived]	1248, 1208	7.70	(6.2–9.5)	1	–	0.1931
2	1290, 1208	6.70	(5.2–8.5)	0.86	(0.61–1.20)	
3	1299, 1116	8.10	(6.5–10.0)	1.04	(0.75–1.45)	
4	1384, 1137	8.70	(7.1–10.7)	1.13	(0.81–1.57)	
[most deprived]	1448, 1086	6.00	(4.6–7.9)	0.76	(0.52–1.10)	
**Academic qualifications** c						
No academic qualifications	890, 887	4.80	(3.4–6.7)	1	–	0.0002
Academic qualifications typically gained at age 16^d^	2313, 2026	6.30	(5.2–7.7)	1.38	(0.91–2.08)	
Studying for/attained further academic qualifications	3179, 2636	9.30	(8.1–10.6)	2.10	(1.39–3.16)	
**Employment status**						
Full‐time employed	2103, 1844	7.10	(5.9–8.6)	1	–	0.0124
Part‐time employed	1755, 1661	5.90	(4.7–7.4)	0.84	(0.62–1.14)	
Unemployed	2374, 1706	8.50	(7.2–9.9)	1.15	(0.89–1.50)	
Retired	415, 524	9.90	(7.3–13.3)	1.79	(1.10–2.90)	
***Health factors***						
**Current depression** e						
No	5885, 5149	7.00	(6.2–7.9)	1	–	0.0002
Yes	780, 602	11.30	(9.0–14.1)	1.68	(1.28–2.21)	
**Self‐reported health status**						
Very good/Good	5683, 4851	6.40	(5.6–7.2)	1	–	<0.0001
Fair	780, 709	13.10	(10.6–16.2)	2.30	(1.74–3.04)	
Bad/Very bad	206, 195	13.30	(8.6–19.9)	2.37	(1.45–3.89)	
**Number of self‐reported chronic conditions**						
0	4357, 3536	6.40	(5.5–7.3)	1	–	<0.0001
1	1544, 1405	7.70	(6.4–9.3)	1.32	(1.02–1.70)	
2+	767, 814	11.80	(9.4–14.6)	2.31	(1.69–3.15)	
**Menopausal status** f						
Not menopausal	5485, 4187	6.70	(6.0–7.6)	1	–	<0.0001
Menopausal	1167, 1548	9.40	(7.7–11.4)	3.20	(2.05–5.00)	

a Unw., unweighted; wtd, weighted.

b Index of Multiple Deprivation (IMD) is a multi‐dimensional measure of area (neighbourhood)‐level deprivation based on the participant's postcode. In Natsal‐3 IMD scores for England, Scotland and Wales were adjusted before being combined and assigned to quintiles, using a method by Payne and Abel to allow use of single Index of Multiple Deprivation measure for the three countries.

c Participants aged ≥17 years.

d English General Certificate of Secondary Education or equivalent.

e Two screening questions (scored 0–3 per question; defined here by a total score of ≥3) assessed depressive symptoms (Patient Health Questionnaire‐2, PHQ‐2), participants were asked whether they had been bothered by feeling down, depressed or hopeless, and whether they had been often bothered by little interest or pleasure in doing things, in the previous 2 weeks.

f Menopausal if woman was older than 45 years and had not had a period in more than a year.

aOR, age‐adjusted odds ratio.

We found strong associations between reporting painful sex and poor health, including overall self‐reported health status (aOR for bad/very bad health versus good/very good 2.37; 1.45–3.89), reporting two or more chronic health conditions (aOR 2.31; 1.69–3.15) and depressive symptoms (aOR 1.68; 1.28–2.21). Reporting painful sex was also positively associated with menopausal status (aOR 3.20; 2.05–5.00).

Table 4 shows associations between reporting painful sex (≥3 months) and selected sexual behaviour, relationship and attitudinal factors. In relation to other aspects of sexual health, experiencing painful sex was associated with feeling like one ought to have known more about sexual matters at first sexual experience (aOR 1.81; 1.36–2.42), and with key adverse sexual health outcomes, specifically reporting an STI diagnosis in the past 5 years (aOR 1.85; 1.27–2.68) and previous experience of non‐volitional sex (aOR 2.17; 1.68–2.80). We found no association with frequency of intercourse (Table 4), or with number of partners reported in the last year (data not shown).

**Table 4:** Sexual behavior, sexual relationship and attitudinal factors associated with reporting painful sex lasting 3 months or more in the last year, among sexually active women aged 16‐74

	Denominators Unw, wtd^a^	Pain during sex for 3 months or more, past year				
		%	95% CI	aOR	95% CI	*P*‐value
All women	6669, 5755	7.50	(6.7–8.3)			
***Sexual behaviour and history***						
**>4 sex acts in last 4 weeks** b						
No (0–4)	4129, 3748	7.50	(6.6–8.5)	1	–	0.3800
Yes (>4)	2078, 1655	6.90	(5.7–8.4)	0.89	(0.69–1.15)	
**Would like to have known more at first sex**						
No	1546, 1326	4.80	(3.7–6.1)	1	–	0.0001
Yes	5097, 4407	8.30	(7.4–9.2)	1.81	(1.36–2.42)	
**Diagnosed with any STI in the past 5 years** c						
No	6232, 5495	7.20	(6.5–8.0)	1	–	0.0012
Yes	397, 228	12.80	(9.4–17.1)	1.85	(1.27–2.68)	
**Ever experienced non‐volitional sex** d						
No	5815, 5055	6.60	(5.9–7.5)	1	–	<0.0001
Yes/Don't know	848, 695	13.40	(10.9–16.3)	2.17	(1.68–2.80)	
***Sexual relationship***						
**Partner does not share the same level of interest**						
No/other	3211, 3064	5.70	(4.8–6.7)	1	–	<0.0001
Yes	1166, 1155	13.40	(11.2–16.0)	2.56	(1.97–3.33)	
**Partner does not have same sexual likes and dislikes**						
No/other	4079, 3908	7.20	(6.3–8.2)	1	–	<0.0001
Yes	297, 310	15.40	(11.2–20.9)	2.35	(1.59–3.47)	
**Partner had sexual difficulties last year**						
No/other	3726, 3498	7.30	(6.3–8.4)	1	–	0.0200
Yes	649, 719	10.10	(7.7–13.1)	1.48	(1.06–2.06)	
**Does not feel emotionally close to partner during sex**						
No/other	4263, 4108	7.70	(6.7–8.7)	1	–	0.0416
Yes	112, 109	13.60	(7.8–22.6)	1.91	(1.03–3.55)	
***Attitudes to sex***						
**Always easy to talk about sex with partners**						
Yes	1746, 1451	5.50	(4.3–6.9)	1	–	0.0017
No/other	4907, 4289	8.10	(7.2–9.1)	1.54	(1.18–2.02)	
**Men have a naturally higher sex drive than women**						
Strongly disagree/disagree/neither agree nor disagree	3351, 2830	6.40	(5.6–7.4)	1	–	0.0053
Strongly agree/agree	3317, 2925	8.40	(7.3–9.7)	1.35	(1.09–1.67)	

a Unw., unweighted; wtd, weighted.

b Defined as vaginal, oral, or anal intercourse.

c Diagnosed with chlamydia, gonorrhoea, herpes, genital warts, trichomonas, non‐specific or non‐gonococcal urethritis, or syphilis but excluding thrush.

d Defined as anyone having sex with you against your will after the age of 13 years.

aOR, age‐adjusted odds ratio.

We did not find associations with relationship status, relationship duration or general happiness with the relationship (data not shown). However, reporting painful sex was strongly associated with sexual aspects of the relationship; including not sharing the same level of interest in sex (aOR 2.56; 1.97–3.33), not sharing the same sexual likes and dislikes (aOR 2.35; 1.59–3.47), and partner having a sexual difficulty (aOR 1.48; 1.06–2.06).

Finally, we found strong associations between reporting painful sex and finding it difficult to talk about sex with partners (aOR for easy versus not always easy 1.54; 1.18–2.02), as well as agreement with the statement that men have a higher sex drive than women (aOR 1.35; 1.09–1.67).

## Discussion

### Main findings

Painful sex lasting ≥3 months in the last year is not uncommon; it is reported by 7.5% of women, of whom one‐quarter (i.e. 1.9% of all sexually active women) report morbid painful sex (symptoms occurring very often or always, symptoms experienced for ≥6 months and fairly or very distressed about the difficulty). The proportion reporting painful sex is highest in young women (16–24 years) and those in later mid‐life (55–64 years), although there was no significant trend with age. Reporting painful sex was strongly associated with experiencing other sexual function problems, notably vaginal dryness, anxiety and lack of enjoyment of sex. The experience of pain was also associated with other aspects of sexual function (including distress and dissatisfaction with sex life, and sexual relationship factors), as well as with adverse sexual experiences such as STI diagnosis and non‐volitional sex, and with indicators of mental and physical health.

### Strengths and limitations

The strength of our study is that it is based on a large national probability sample with a wide age range.^20^, ^21^ We achieved a response rate in line with other major social surveys in Britain^22^ and higher than many other population prevalence studies of sexual function.^23^, ^24^ A limitation is that our measurement of pain was based on a single item, rather than on clinical diagnosis; however, obtaining clinically sufficient information is rarely possible in general population surveys,^25^ and would be particularly challenging here given the lack of consensus on aetiology and classification. Nonetheless, it is worth bearing in mind that the causes of painful sex included in our sample may be wide ranging. It is likely that we have slightly underestimated the prevalence of painful sex because our figures include only sexually active women; of women who were ever sexually active but not in the last year, 2.49% said they had avoided sex because of pain, or fear of feeling pain. [Correction added on 7 April 2017, after first online publication: The percentage calculation has been corrected from 2.05% to 2.49% in the preceding sentence due to a mistake in the calculation in the denominator.] Missing from this estimate is 150 women who had never had a sexual experience and were not directed to the self‐complete section of the questionnaire Another limitation is that, because of the cross‐sectional nature of the data, we are unable to infer causality. Sexual pain may cause, stem from, or co‐exist with poor psychosocial, physical and sexual health, and even for in‐depth research, the challenge of delineating causal pathways is daunting. As with almost all research on pain, we relied on self‐report data. We sought to minimise reporting bias by including items on pain in the self‐complete section of the questionnaire.

### Interpretation

Prevalence studies vary in measurement and sampling approaches, limiting the value of comparison across population‐based studies.^4^ Our estimates can be compared most reliably with studies using probability sampling and one broad item to measure pain. In the US National Health and Social Life survey, Laumann et al. reported prevalence of pain during sex ranging from 21% among women aged 18–29 years to 8% among women aged 50–59 years.^26^ The Australian Longitudinal Study of Health and Relationships, measuring painful sex lasting ≥1 month in the past year, estimated a prevalence of 10% among women aged 20–64 years.^27^ Our estimates were slightly lower than this, and decreased further when stricter morbidity criteria were used (see also ref. 28), and this effect has also been found in other population surveys.^24^, ^29^ Other studies do not measure dyspareunia per se, but report estimates of genital pain disorders such as vulvodynia. Studies using telephone surveys to ask about detailed symptomatology generally produce estimates of around 3.8%^17^ and 4.2%^5^ for vulvar pain of ≥6 months duration, and 8.3% for vulvodynia.^30^

Our data confirm the strong link between pain and impaired sexual function found in the literature.^2^, ^7^, ^12^, ^31^ The relationship context of pain is beginning to receive more attention,^7^ but evidence of the link between sexual pain and relationship adjustment and satisfaction is equivocal.^32^ We found no association between sexual pain and general happiness in the relationship, but strong associations with sexual aspects of the relationship, such as not sharing the same level of interest in sex, not feeling emotionally close, not sharing the same sexual likes and dislikes, and difficulty talking about sexual matters. This is consistent with the conclusion of a recent review, suggesting that while sexual pain is associated with sexual dissatisfaction, it is not necessarily associated with poor relationship adjustment in general. 32

A number of studies have linked childhood abuse with later experience of painful sex.^12^, ^33^ We found a link with sex against one's will, after the age of 13 years, suggesting that nonvolitional sex in adolescence and adulthood is also important. STIs are a known direct cause of genital pain^34^ and although this may explain the association we found, it is also possible that previous experience of an STI leads some individuals to associate sex with disease, possibly engendering difficulties with arousal, and in turn, leading to painful sex.^2^ In contrast to previous research,^7^, ^12^, ^31^ we did not find an association of pain with frequency of intercourse. It has been suggested that some women simply endure their symptoms in order to continue having sex with their partner^2^, ^35^ and our inclusion of women with milder symptoms (compared with clinical samples) may have resulted in a higher proportion of women who continue to have sex despite their symptoms. The Natsal‐3 survey found that people also have sex less frequently nowadays,^21^ making it harder to detect a significant association with frequency.

Our finding of a clear association between pain and self‐reported health and reporting two or more chronic health conditions is consistent with the literature linking pain with a range of chronic conditions such as irritable bowel syndrome, urinary tract infections, depression and chronic fatigue syndrome.^12^, ^17^, ^36^ In postmenopausal women, painful sex is typically associated with dryness resulting from vaginal atrophy.^37^ We found that reporting painful sex was associated with menopausal status, although several studies have failed to find such an association.^5^, ^17^, ^38^ Our finding of an association with depressive symptoms is supported by case–control studies^7^, ^12^, ^14^ but has not always been found in population studies.^15^, ^31^

Although painful sex is less commonly reported by women than lacking interest in sex and difficulty reaching climax,^39^ it is the sexual function problem most commonly experienced as distressing.^28^ It can lead to feelings of isolation, shame, sexual inadequacy, loss of confidence and feeling out of control.^7^, ^8^, ^38^ Only a fraction of women affected by genital pain disorders ever receive an official diagnosis: 1.4% in a study of women meeting criteria for vulvodynia.^30^ In a previous paper from Natsal‐3 28 we reported that less than half of women with morbid symptoms of sexual pain had sought professional help in the last year. Among those who do seek treatment, negative experiences are common, including invalidated concerns, not receiving a formal diagnosis, and being given treatment perceived as ineffective.^5^, ^40^

## Conclusion

### Practical and research recommendations

In terms of clinical assessment, our data support the literature in demonstrating the importance of taking a holistic and detailed history: thoroughly investigating symptoms, asking about enjoyment and satisfaction, and taking into account the relationship context. There is a need for resources to support clinicians who feel uncomfortable broaching the topic of sexual function and pleasure with their patients, including advice on language and on when to refer patients to specialists in sexual health.

We have confirmed the strong link between painful sex and self‐assessed poor health and experience of chronic health conditions. Further research might explore the extent to which painful sex might usefully serve as a diagnostic indicator of other health problems, in the same way that erectile difficulties often signify cardiovascular problems in men.^41^

Our finding of an association between painful sex and wanting to have known more at first sexual experience has been demonstrated elsewhere.^33^ Given that painful sex is common in younger women, and that half of young women report their first experience of intercourse as painful,^42^ it would seem prudent to ensure that the possibility of pain is discussed openly in sex education and in consultations between young people and health professionals.

This study provides up‐to‐date prevalence estimates of painful sex in a representative sample of British women, across a wide age range. It is also rare in exploring—simultaneously and in detail—associations between dyspareunia and sexual functioning of the relationship, previous sexual history, attitudes towards sex and general health. In doing so, it has addressed a gap in the understanding of the social and relationship patterning of painful sex (dyspareunia) at population level. Our findings are important and relevant to the work of a range of practitioners involved in gynaecology, oncology, psychosexual therapy, and more broadly in therapeutic settings.

### Disclosure of interests

AM Johnson has been a Governor of the Wellcome Trust since 2011. The remaining authors have nothing to disclose. Completed disclosure of interests form available to view online as supporting Information.

### Contribution to authorship

The paper was conceived by KRM, CHM, RG, KGJ, CG, DN and JB. KRM wrote the first draft, with further contributions from all authors. Statistical analyses were undertaken by RG, CHM and KGJ. CHM, AMJ (Principal Investigator), PS and KW, initial applicants on Natsal‐3, wrote the study protocol and obtained funding. Natsal‐3 questionnaire design, ethics applications and piloting were undertaken by KRM, CHM, AMJ, PS, NF, JD and KW. Data management was undertaken by NatCen Social Research, UCL and LSHTM. All authors contributed to data interpretation, reviewed successive drafts and approved the final version of the manuscript.

### Details of ethics approval

Natsal‐3 was approved by the NRES Committee South Central—Oxford A (Ref: 10/H0604/27) on 12 July 2010. Participants provided oral informed consent for interviews.

### Funding

Natsal‐3 was supported by grants from the Medical Research Council [G0701757] and the Wellcome Trust [084840] with contributions from the Economic and Social Research Council and Department of Health. Since September 2015 KRM has been core funded by the UK Medical Research Council through the MRC/CSO Social & Public Health Sciences Unit, University of Glasgow [MC_UU_12017‐11]. The sponsors played no role in the study design, data interpretation, data collection, data analysis or writing of the report. The corresponding author had full access to all the data in the study and had final responsibility for the decision to submit for publication.

## Supporting information


**Video S1.** Author Insights.
